# Development of a Tetracycline-Inducible System for Conditional Gene Expression in Lactococcus lactis and Streptococcus thermophilus

**DOI:** 10.1128/spectrum.00668-23

**Published:** 2023-05-16

**Authors:** Sofia Markakiou, Ana Rute Neves, Ahmad A. Zeidan, Paula Gaspar

**Affiliations:** a R&D Department, Chr. Hansen A/S, Hørsholm, Denmark; b Department of Biochemistry, University of Groningen, Groningen, Netherlands; National Research Council of Italy

**Keywords:** lactic acid bacteria, *Lactococcus lactis*, *Streptococcus thermophilus*, inducible promoter, tetracycline, markerless mutagenesis, genetic engineering, linear DNA editing fragment, DNA assembly

## Abstract

Inducible gene expression systems are invaluable tools for the functional characterization of genes and in the construction of protein overexpression hosts. Controllable expression is especially important for the study of essential and toxic genes or genes where the level of expression tightly influences their cellular effect. Here, we implemented the well-characterized tetracycline-inducible expression system in two industrially important lactic acid bacteria, Lactococcus lactis and Streptococcus thermophilus. Using a fluorescent reporter gene, we show that optimization of the repression level is necessary for efficient induction using anhydrotetracycline in both organisms. Random mutagenesis in the ribosome binding site of the tetracycline repressor TetR in Lactococcus lactis indicated that altering the expression levels of TetR was necessary for efficient inducible expression of the reporter gene. Through this approach, we achieved plasmid-based, inducer-responsive, and tight gene expression in Lactococcus lactis. We then verified the functionality of the optimized inducible expression system in Streptococcus thermophilus following its chromosomal integration using a markerless mutagenesis approach and a novel DNA fragment assembly tool presented herein. This inducible expression system holds several advantages over other described systems in lactic acid bacteria, although more efficient techniques for genetic engineering are still needed to realize these advantages in industrially relevant species, such as S. thermophilus. Our work expands the molecular toolbox of these bacteria, which can accelerate future physiological studies.

**IMPORTANCE** Lactococcus lactis and Streptococcus thermophilus are two industrially important lactic acid bacteria globally used in dairy fermentations and, therefore, are of considerable commercial interest to the food industry. Moreover, due to their general history of safe usage, these microorganisms are increasingly being explored as hosts for the production of heterologous proteins and various chemicals. Development of molecular tools in the form of inducible expression systems and mutagenesis techniques facilitates their in-depth physiological characterization as well as their exploitation in biotechnological applications.

## INTRODUCTION

Lactic acid bacteria (LAB) are a heterogeneous but functionally related group of Gram-positive bacteria, characterized by the production of lactic acid as the main end product of their carbohydrate metabolism. They have been applied extensively in food fermentations, where they provide natural preservation and contribute to flavor and texture formation (1).

Because of its industrial importance, the most widely used species of LAB, Lactococcus lactis, has been extensively studied over the past decades. This has generated a wealth of information regarding its unique metabolic processes and motivated the development of molecular tools allowing for its extensive physiological characterization (2). The food-grade status, thoroughly studied metabolism, and genetic amenability have made L. lactis an attractive host for the production of chemicals and enzymes, as well as a promising drug and vaccine delivery agent in biotherapeutic applications (3, 4). Although equally important in dairy fermentations, the metabolic potential of Streptococcus thermophilus has only recently been recognized (5, 6). This bacterium is traditionally used for yogurt fermentation in coculture with Lactobacillus delbrueckii subsp. *bulgaricus* and in the production of several cheese varieties, such as cheddar. Applications beyond food fermentation are currently hampered by the lack of sophisticated tools for efficient genetic manipulation (6).

Expanding our understanding of the physiology and exploring opportunities for new applications for L. lactis and S. thermophilus require efficient molecular tools for gene perturbation, construction of reporter strains, and controllable gene expression. Inducible gene expression systems are particularly relevant for the functional characterization of gene products in the cellular environment, the heterologous overexpression of proteins, and the design of complex regulatory gene circuits (7). Many controllable promoters have been described for L. lactis, such as the extensively used nisin-inducible promoter P*_nisA_* (8) and several others that are sensitive to environmental stimuli, namely, low-pH (9) and stress conditions (10), or are inducible by sugars (11), metals (12, 13), salts (14), phages (15, 16), or agmatine (17; also reviewed in references 2, 18, and 19). However, almost 20 years after its discovery, induction by nisin remains the most popular solution for L. lactis, due to the high expression levels that can be achieved and the tight control offered by the system under most conditions.

In S. thermophilus, inducible expression systems are scarce and, most often, poorly characterized. The promoter P*_nisA_* from L. lactis has been used to control the heterologous expression of the pediocin operon (20, 21), while the novel promoter P*_stbD_*, controlled by a peptide pheromone (22), has been used for the induction of ComX, with medium-dependent leakiness being observed (23). Additional promoters controlled by molecules or environmental stimuli have been described for S. thermophilus, responding to lactose (24), the inducer peptide ComS (25), acid (26) or heat stresses (27). However, most of them either show considerable levels of leaky expression or have not been extensively characterized in terms of level and dynamic response of induction.

One of the most utilized transcriptional regulators for controllable gene expression in both prokaryotic and eukaryotic organisms is the tetracycline repressor TetR (7), which is found in several bacterial species, where it controls genes related to tetracycline resistance (28). In principle, TetR forms homodimers that bind to its cognate DNA binding site, *tetO*. Through this action, TetR exerts transcriptional repression on genes containing *tetO* sites inside or in close proximity to their promoters, as this prohibits binding of the RNA polymerase. In the presence of tetracycline (or a less toxic analogue, such as anhydrotetracycline [ATC]), TetR undergoes conformational changes due to its interaction with these molecules, which consequently prevents binding to the *tetO* site and permits transcription of the target gene. In Gram-positive bacteria, systems for inducible gene expression based on TetR and its cognate DNA binding site, *tetO*, have been employed successfully in Bacillus subtilis (29), Staphylococcus aureus (30), and Streptococcus pneumoniae (31).

Tools for genetic engineering in S. thermophilus are also limited compared to those for other well-studied bacteria. Plasmid-based integration systems were initially applied, despite inherent disadvantages, such as the introduction of polar effects in the genome (6). Upon discovery of natural competence in S. thermophilus (23), linear DNA-based systems became the method of choice for genome engineering in this microorganism. The simplest approach relies on linear DNA fragments consisting of an antibiotic resistance gene flanked by locus-specific homologous regions, which upon integration result in genomic editing as well as permanent antibiotic resistance, which may have unintended adverse metabolic effects. This also restricts the number of mutations that can be introduced to a single strain due to the limiting availability of antibiotic selection markers. Fontaine et al. (32) improved this approach by introducing *loxP* sites flanking the antibiotic selection marker, permitting its excision by a plasmid-expressed Cre recombinase after integration. This leads to markerless editing, although a *lox72* scar remains and subsequent plasmid curing becomes necessary. A more recent approach employs linear DNA fragments containing both an antibiotic resistance gene and the counterselection gene *oroP* from L. lactis, requiring two integration events for markerless deletion (33). Following the integration of the first DNA fragment, a second linear DNA fragment containing the desired edit and targeting the same locus is introduced. Selection for the loss of the *oroP* gene promotes integration of the second fragment, resulting in a markerless mutation (34).

In this study, we describe the development of an optimized tetracycline-controlled gene induction system through fine-tuning the expression levels of the tetracycline repressor TetR. The functional properties of the expression system were studied in L. lactis and S. thermophilus using the fluorescent protein mCherry as a reporter. Furthermore, a markerless mutagenesis approach, which requires only a single transformation step and relies on linear DNA editing fragments containing a counterselection marker, was applied in S. thermophilus. To facilitate the construction of the necessary multicomponent editing fragments, we additionally developed a dual-vector Linear DNA Editing Fragment (LDEF) tool, allowing for efficient and reproducible fragment assembly through Golden Gate cloning.

## RESULTS AND DISCUSSION

### Design of a tetracycline-inducible expression system for lactic acid bacteria.

**FIG 1:**
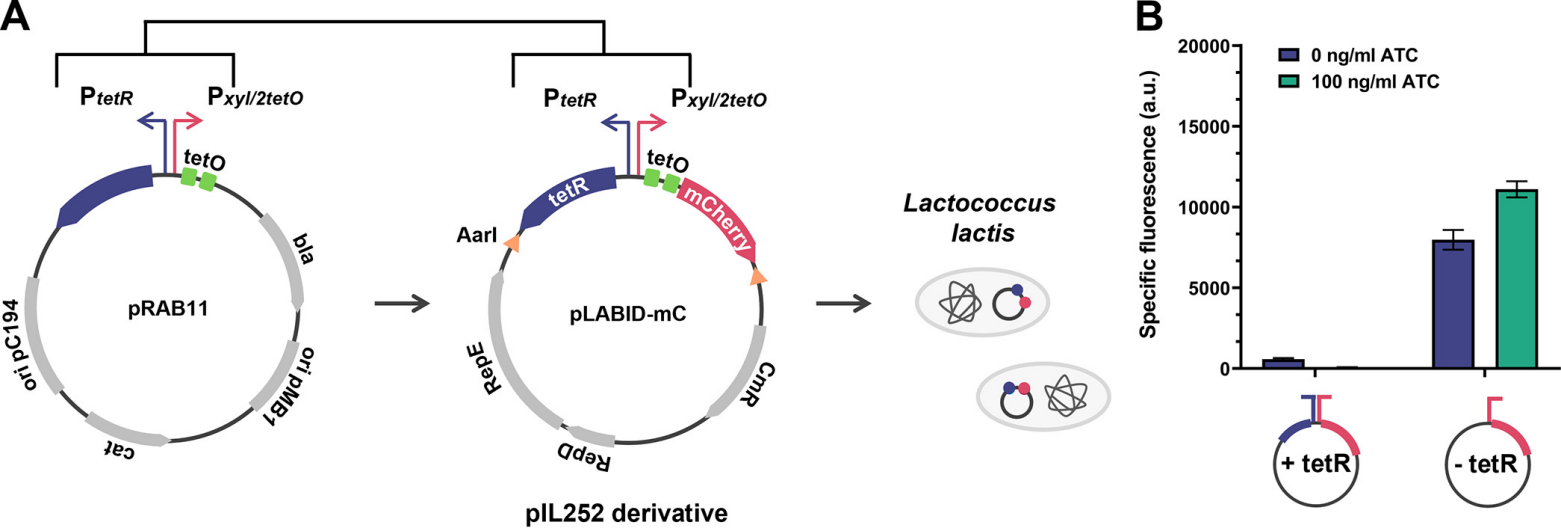
Vector for tetracycline-inducible gene expression in LAB. (A) Genetic map of pLABID-mC, derivative of the widely used and broad-host-range vector pIL252. The fragment *tetR*-P*_xyl/2tetO_* from the staphylococcal vector pRAB11 was introduced to pIL252CmR. Insertion of the *mCherry* gene downstream of promoter P*_xyl/2tetO_* allows for direct evaluation of repression efficiency by TetR. Thick arrows represent genes, and squares represent the *tetO* operators, while a bent arrow indicates a promoter. Orange triangles represent AarI restriction sites, permitting the use of the vector in Golden Gate cloning. (B) Efficiency of mCherry induction from pLABID-mC in L. lactis MG1363. Expression of TetR in pLABID-mC (+*tetR*) leads to permanent repression of promoter P*_xyl/2tetO_* even in the presence of the inducer ATC. Deletion of *tetR* in pLABID-mC-ΔtetR (−*tetR*) reverses this phenotype, reconstituting *mCherry* expression.

To develop a simple and yet efficient inducible expression system that can be applied in both L. lactis and S. thermophilus, we relied on the documented adaptability of the *tet* system in bacteria (7). Towards this, and using L. lactis as a first host due to its greater genetic amenability, the control module (*tetR*-P*_xyl/2tetO_*) from plasmid pRAB11 (35), an optimized variant of pRMC2 (36), was transferred to a chloramphenicol-resistant variant of the widely used, low-copy-number, and broad-host-range LAB vector pIL252 (37), generating the vector pLABID (Fig. 1A). The P*_xyl/2teO_* promoter of this module relies on the xylose operon promoter P*_xylA_* of B. subtilis (29), with two tetracycline operator sites introduced (29, 35) and the xylose operator site removed (38), allowing for tetracycline-inducible but xylose-independent gene expression at high levels.

To test the functionality of pLABID using a simple and direct readout, the *mCherry* variant of pEFB001, codon optimized for low-GC-content LAB (39), was positioned under the promoter P*_xyl/2tetO_*, resulting in plasmid pLABID-mC. Induction in L. lactis MG1363 using 100 ng mL^−1^ anhydrotetracycline (ATC), the highest concentration of the inducer not resulting in growth retardation (see Fig. S1 in the supplemental material), did not lead to significant mCherry expression, as evidenced by the absence of a fluorescence signal (Fig. 1B). To investigate the lack of system functionality, the compatibility of the B. subtilis hybrid P*_xyl/2tetO_* promoter with L. lactis was assessed. For this, the *tetR* gene was deleted from pLABID-mC, resulting in plasmid pLABID-mC-ΔtetR. This derivative displayed high mCherry expression independently of the inducer’s presence (Fig. 1B), suggesting that the cellular concentration of TetR rather than the promoter P*_xyl/2tetO_* was responsible for the absence of mCherry production in pLABID-mC. As excessive expression of a transcriptional repressor can lead to a constitutively repressed system (40, 41), it was hypothesized that the *tet*-responsive promoter P*_xyl/2tetO_* in pLABID-mC was always strongly repressed, inhibiting mCherry expression even in the presence of maximum inducer concentrations.

These results show that transcriptional repression of genes under the control of the promoter P*_xyl/2tetO_* is functional in L. lactis, although optimization of the TetR repressor levels is necessary. Module/host compatibility is a frequently encountered issue and limitation in synthetic biology, often making the optimization of genetic systems through targeted or random approaches necessary before their implementation in other organisms (42). Fine-tuning of the tetracycline-inducible system was also required in the Gram-positive S. aureus, by increasing the expression of TetR (36) or semirationally engineering the promoter P*_xyl/2tetO_* (35), in order to improve the repression and leakiness levels.

### Optimization of TetR levels is necessary for mCherry induction by ATC in L. lactis.

**FIG 2:**
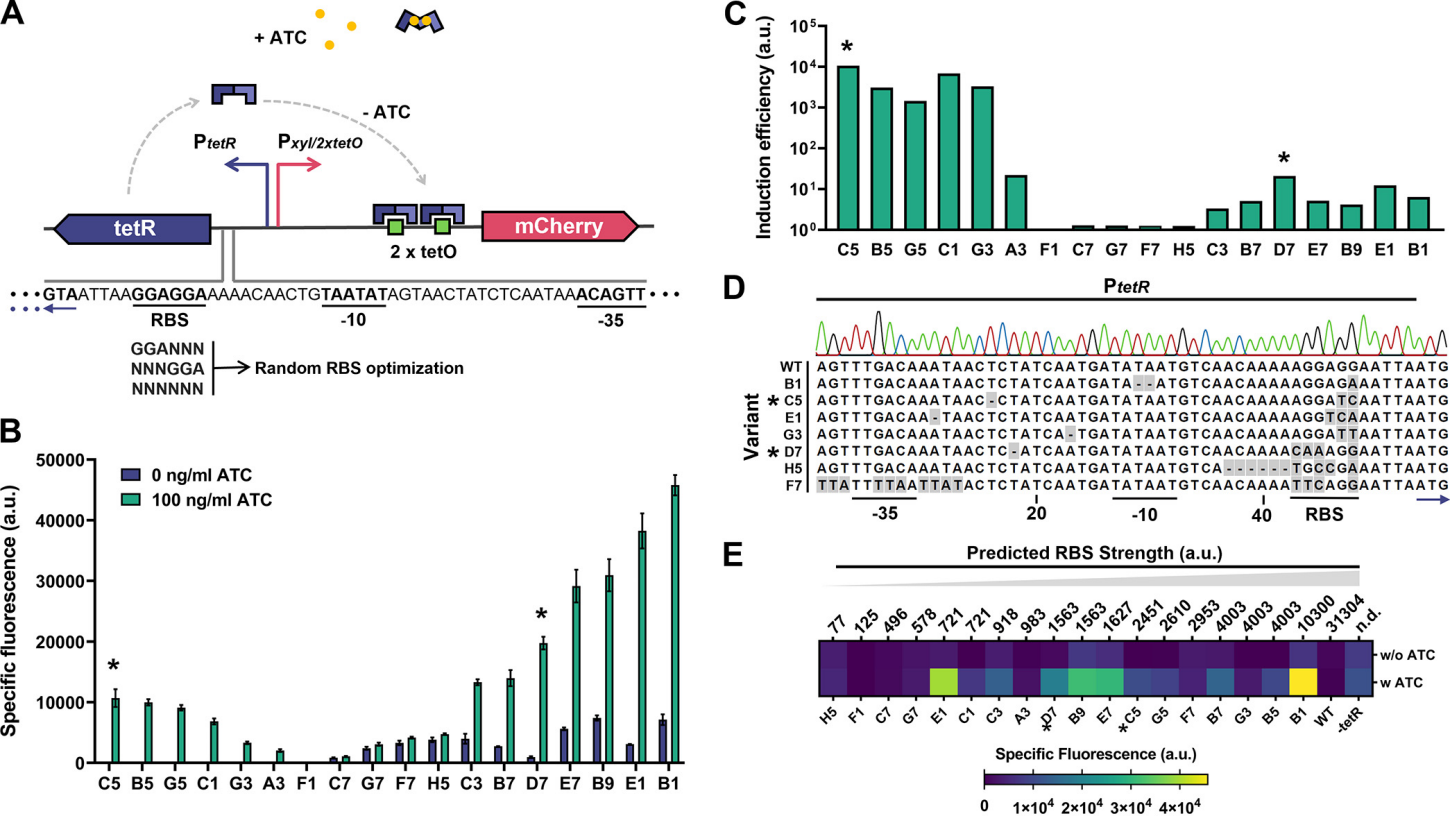
Optimized TetR repression in pLABID-mC through random mutagenesis in L. lactis. (A) Schematic representation of the *tet* regulatory system in pLABID-mC. Genes, operators, and promoters are represented by thick arrows, squares, and bent arrows, respectively. TetR, constitutively expressed by P*_tetR_*, binds as a dimer to the *tetO* operators incorporated in the P*_xyl/2tetO_* promoter, repressing expression of *mCherry*. In the presence of ATC (yellow dots), TetR binding to *tetO* sites is inhibited, permitting *mCherry* expression. To allow for derepression of *mCherry* expression by TetR, the RBS site of the depicted P*_tetR_* promoter was modified by random mutagenesis using degenerate oligonucleotides. (B) Specific fluorescence of L. lactis MG1363 cells harboring different variants of pLABID-mC in the absence and presence of ATC, after overnight cultivation. a.u., arbitrary units. (C) Induction efficiency of selected pLABID-mC variants in L. lactis, calculated as the ratio of the average specific fluorescence in the presence of ATC against the average specific fluorescence in the absence of ATC. (D) Mutation analysis for selected pLABID-mC variants. Letters or dashed lines marked with gray represent deviations from the original sequence of P*_tetR_*, here indicated as WT. The blue arrow indicates the starting codon of *tetR*. (E) Plot of predicted RBS strength for the selected variants against the observed specific fluorescence in the absence or presence of 100 ng mL^−1^ ATC. Variants labeled with an asterisk were selected for further characterization.

To permit for transcriptional induction of *mCherry* in pLABID-mC, optimization of the intracellular levels of TetR was necessary, as indicated above. For this, we applied a similar approach to the one described by Siedler and coworkers (41), by performing random mutagenesis on the ribosome binding site (RBS) of *tetR* (Fig. 2A). In bacteria, initiation of protein translation is considered a rate-limiting step, and modification of the RBS by changing only a few bases can be enough for modulating the translation level of a protein (43).

After random mutagenesis on the RBS of *tetR* in pLABID-mC, the resulting bacterial library was screened for mCherry expression in the presence and absence of ATC in agar plates. From the ~600 colonies screened, the predominant population showed low or no detectable levels of fluorescence under both conditions, indicating a still prohibitive TetR expression level. Other colony variants exhibited mCherry expression with and without ATC, presumably due to inefficient repression of the P*_xyl/2tetO_* promoter. However, a few promising variants were identified, showing low or no fluorescence in the absence of ATC, but significantly higher fluorescence values in the presence of the inducer (Fig. 2B). From the 18 selected clones, variant C5 displayed the highest inducible mCherry expression with no detectable basal expression and variant D7 exhibited a 20-fold induction in the presence of the inducer with minimal basal expression, making them the candidates with the most promising phenotypes (Fig. 2C). Variant F1, showing no detectable fluorescence level, and variants C7, G7, F7, and H5, displaying a steady fluorescence signal irrespective of the presence of the inducer, were included as controls for further analyses.

To understand how the levels of TetR modulate P*_xyl/2tetO_* induction, we sequenced the selected clones included in Fig. 2B and used the RBS Calculator (44), a tool that predicts the theoretical translation initiation rate of mRNAs in bacteria, to correlate the presence of specific RBS mutations to the translation level of TetR and, subsequently, to the level of mCherry repression. Surprisingly, sequencing revealed additional mutations in regions other than the targeted 6-bp-long RBS site of *tetR*, mainly in the spacer sequence between the −10 and −35 regions of the *tetR* promoter (Fig. 2D; Fig. S2). The presence of these mutations could be attributed to the use of poor-quality degenerate oligonucleotides during mutagenesis or to the occurrence of a selection pressure favoring their appearance, assuming that they can affect the strength of the *tetR* promoter. Comparative growth of L. lactis MG1363 harboring pIL252CmR, pLABID-mC, or no plasmid at all indicates that expression of pLABID-mC negatively affects cell fitness, as evidenced by a reduction in the growth rate and final biomass for cells expressing this plasmid compared to the backbone vector pIL252CmR (Fig. S3). As *mCherry* is constitutively repressed in pLABID-mC, this burden can only be associated with the expression of TetR. This could suggest that the additional *tetR* promoter mutations are a form of stress adaptation to the expression of TetR. However, these mutations appeared in all but two clones (A3 and C7), irrespective of the inducibility of P*_xyl/2tetO_*. Assuming that the inducibility of P*_xyl/2tetO_* correlates to the levels of TetR, a stress adaptation does not explain the presence of *tetR* promoter mutation in variants sharing a similar phenotype to pLABID-mC, such as F1. This indicates that the occurrence of these promoter mutations is more likely a stochastic event, rather than a driven response.

Nevertheless, similarly to base alterations in the −10 and −35 promoter regions (45), changes in spacer length and composition can have a significant effect on promoter strength (46, 47). This suggest that the final intracellular amount of TetR is most likely adjusted on both the translational and transcriptional levels in the derivatives of pLABID-mC. Due to this, correlation of the predicted *tetR* RBS strength to the induction level of mCherry (Fig. 2E) was inevitably inconclusive in estimating an optimal range of RBS strength for the former. As an example, clones with similar induction profiles, such as E1 and B1, display largely dissimilar theoretical RBS strengths, but both contain additional base pair alterations in the core promoter region of *tetR* (Fig. 2D), suggesting that any changes in TetR levels result from the combined effect of modifications at the transcriptional and translational levels. Based on these pieces of evidence, further experiments, such as the quantification of the *tetR* transcripts or resulting protein levels, would be required in order to derive a possible correlation.

In summary, by applying random mutagenesis to optimize the levels of the tetracycline repressor TetR, we were able to generate ATC-responsive variants of pLABID-mC, although we could not to devise a correlation between TetR expression levels and P*_xyl/2tetO_* inducibility. This is likely due to transcriptional and translational changes that act synergistically to modulate the expression of TetR in these variants as a combined result of mutations in both the *tetR* promoter and RBS. Despite this, our data still indicate that fine-tuning of the TetR levels is necessary to allow for *mCherry*-inducible expression in the presence of ATC from pLABID-mC. These findings corroborate the need for partial optimization across different genetic backgrounds (48), an issue at the forefront of synthetic biology.

### Markerless genomic integration of the *tetR*-P*_xyl/2tetO_*-*mCherry* cassette in S. thermophilus using linear DNA.

Unlike the well-studied L. lactis, genetic tools for the engineering of S. thermophilus, such as reliable inducible expression systems, are still lacking. Consequently, this hampers genetic engineering-based physiological studies aiming at elucidating the molecular mechanisms underlying some of the key industrial properties of S. thermophilus, such as exocellular polysaccharide production and its influence on texture formation in fermented milk (49–51).

To test the optimized tetracycline-inducible system in S. thermophilus, pLABID-mC and two of its best-performing variants, pLABID-mC-C5 and pLABID-mC-D7, were introduced into the industrially relevant strain ST6 by electroporation. However, all three plasmids, including the empty vector pIL252CmR, proved segregationally unstable under chloramphenicol selection in the absence of ATC induction. Therefore, they were all reconstructed to express the *ermR* gene for erythromycin resistance instead of the chloramphenicol resistance gene *cmR*. This resulted in stable propagation in S. thermophilus ST6 after multiple passages in erythromycin-containing agar plates, but no fluorescence could be observed for these transformants even under conditions of maximum induction with 100 ng mL^−1^ ATC. More careful evaluation of transformants using fluorescence microscopy showed that only a minimal number of cells were expressing mCherry at low levels, an indicator of unstable plasmid expression, despite the low copy number of pIL252 (37), which is unlikely to cause an energetic burden due to protein overexpression. Plasmid-host compatibility can significantly vary from species to species, even among strains of the same species, and often industrially robust, non-laboratory strains may prove more refractory toward stable plasmid propagation and protein expression. Additionally, native plasmids are rare among S. thermophilus strains and usually of a relatively small size (52), which could indicate a general recalcitrance toward plasmid maintenance.

**FIG 3:**
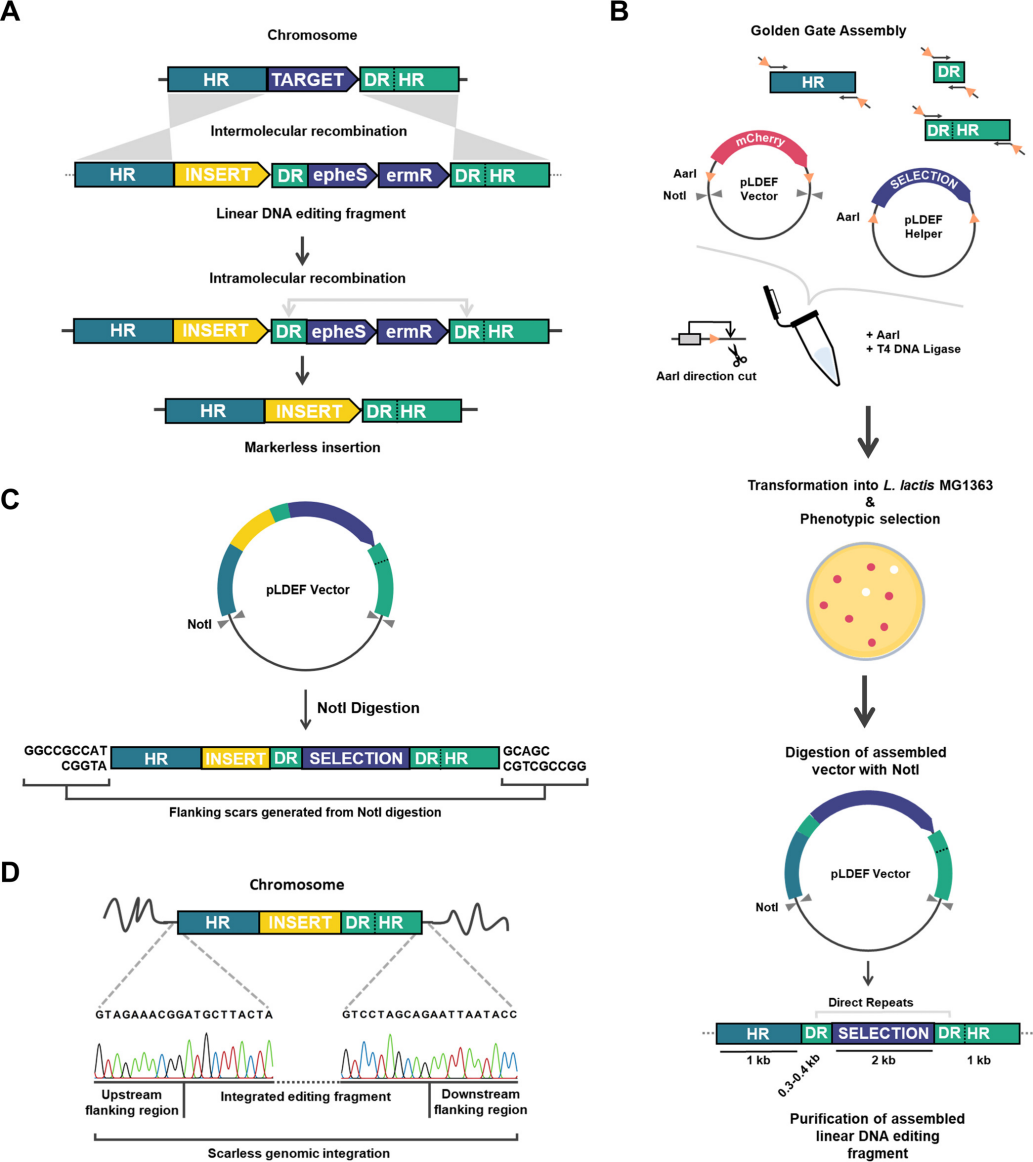
Gene insertion or deletion in S. thermophilus using linear DNA editing fragments. (A) Illustration of chromosomal insertion in S. thermophilus. A linear DNA editing fragment consisting of a standard selection module (P*_ldh_-epheS-ermR*, SELECTION), the desired insert (INSERT), and two direct repeats (DR), all flanked by two target-specific homologous regions (HR), is introduced to S. thermophilus by natural transformation. From the two direct repeats, one is already part of the downstream homologous region. Through homologous recombination under erythromycin selection, the target is replaced by the selection cassette, insert, and one direct repeat. Selection marker excision is achieved via intramolecular recombination under PCPA selection, utilizing the double direct repeats. As the direct repeats are derived from the 5′-end sequence of the downstream homologous region, this excision is scarless. This approach results in a markerless gene insertion and can be adapted for other applications, such as gene deletions and base alterations. (B) Schematic representation of the designed dual-vector LDEF system for generation of linear DNA editing fragments, in this case as indented for a gene deletion. In a one-pot Golden Gate reaction, pLDEF Vector (chloramphenicol resistance), pLDEF Helper (erythromycin resistance), and PCR-amplified fragments corresponding to the locus-specific flanking regions (HR) and direct repeat (DR) are combined. The second direct repeat needed for intramolecular recombination is already part of the downstream homologous region. LDEF Vector acts as scaffold for fragment assembly, while pLDEF Helper provides the *P_ldh_-epheS-ermR* selection module. Through the action of AarI and T4 DNA ligase, the locus-specific DNA editing fragment is assembled in pLDEF Vector, in the region previously occupied by *mCherry*. The absence of *mCherry* allows for phenotypic colony selection by the naked eye as colonies containing the assembled editing DNA fragment appear white, while colonies expressing the undigested pLDEF Vector have a pink color that is visible without the use of a fluorescence illuminator. After assembly, digestion of the pLDEF Vector with NotI releases the now linear DNA editing fragment, which can be directly introduced to S. thermophilus by natural transformation. AarI restriction sites are represented by orange triangles, with their direction indicating the site of AarI digestion. Gray triangles represent NotI restriction sites. (C) Release of the assembled editing fragment from pLDEF Vector by NotI digestion results in 9-bp-long flanking scars, partially corresponding to the used NotI recognition site 5′-GCGGCCGC-3′. (D) Sanger sequencing for S. thermophilus strain ST6-mC, verifying successful integration of the linear DNA editing fragment, without integration of the flanking scars generated by NotI.

As plasmid pLABID-mC and its variants proved segregationally unstable in S. thermophilus ST6, genomic integration of the *tetR*-P*_xyl/2tetO_*-*mCherry* expression cassette was pursued instead. Integration into the genome offers greater stability than plasmid-based expression systems due to the absence of plasmid segregation issues and copy number variation, making it better suited for tight expression control, when needed. This could be of significance when studying the effect of a protein on cell physiology in a dose-dependent manner. To integrate the tetracycline-inducible gene expression system in the genome of S. thermophilus, the method described by Zhang et al. (53) for Streptococcus mutans was applied. This simple yet elegant approach, based on a single transformation event, leads to markerless mutations without leftover scars. As illustrated in Fig. 3A, S. thermophilus strain ST6 was transformed with a linear DNA editing fragment consisting of two 1-kb locus-specific homologous regions flanking the insert (*tetR*-P*_xyl/2tetO_*-*mCherry*), a selection cassette (P*_ldh_*-*epheS*-*ermR*), and an ~300-bp-long direct repeat, which shares a similar sequence with the 5′ end of the downstream homologous region. As an insertion site, the glucose phosphoenolpyruvate-dependent phosphotransferase pseudogene region was used, as previously documented (54). The selection module contained a mutated version of the conserved phenylalanyl-tRNA synthetase alpha subunit (*epheS*) for negative selection and an erythromycin resistance gene (*ermR*) for positive selection, both under the strong promoter P*_ldh_* of the lactate dehydrogenase from S. thermophilus strain ST6. Upon positive selection with erythromycin, the flanking homologous regions facilitate intermolecular recombination with the bacterial chromosome, resulting in integration of the linear DNA editing fragment. Subsequent negative selection using 4-chloro-dl-phenylalanine (PCPA) allows for selection of clones where intramolecular recombination between the direct repeat sequences flanking the selection cassette has occurred, resulting in its excision and, thus, a markerless insertion. As the sequence of the two direct repeats derives from the genome, the insertion is also scarless. In addition, the selection markers are eliminated during the mutagenesis, permitting successive editing rounds in the same strain, if desirable. Although we applied this method for inserting the *tetR*-P*_xyl/2tetO_*-*mCherry* cassette, it can be utilized for genomic deletions or nucleotide substitutions as well.

To apply the counterselection marker *epheS* to S. thermophilus, we first introduced two amino acid mutations to the native protein sequence of strain ST6. Mutation A314G allows for efficient misincorporation of the toxic phenylalanine analogue PCPA to newly synthesized proteins during translation (55), while mutation T260S allows for higher PCPA incorporation efficiency and thus selectivity (56). To maximize the compatibility of the *epheS* counterselection marker with S. thermophilus, we opted for mutagenizing the endogenous wild-type (WT) *pheS* gene from strain ST6 (Fig. S4), as the need for species-specific *epheS* variants has been previously reported (57), despite the high conservation level of PheS among bacteria (Fig. S5).

As this approach relies on chromosomal intermolecular recombination with linear DNA fragments, the ability of various S. thermophilus strains to internalize linear DNA via natural transformation may be limiting. Some bacterial species show very high levels of natural competence, especially when synthetic inducer peptides (58) or strains overexpressing competence-related genes are employed (59–61). Natural competence levels can vary tremendously among S. thermophilus strains though, with many of them remaining recalcitrant. Addition of the synthetic competence peptide inducer ComS_17–24_ can mitigate this issue to a certain degree (32), but efficiencies fluctuate significantly, as corroborated by our personal experience with many industrial strains.

### Design features of the dual-vector LDEF tool for the assembly of linear DNA editing fragments.

As showcased above, genetic engineering through targeted integration of linear DNA to the host’s genome often requires the prior construction and assembly of multicomponent linear DNA editing fragments, a task that can be challenging depending on the number of parts to be assembled, their sequence composition, and length. Therefore, to accelerate genetic manipulation of S. thermophilus using the adapted method described here, we designed and constructed the complementary dual-vector LDEF tool to facilitate assembly of the necessary linear DNA editing fragments using L. lactis as the host.

The tool consists of two plasmids, pLDEF Vector and pLDEF Helper, both derivatives of the broad-host-range vector pNZ8148 (62). The pLDEF Vector plasmid acts as a scaffold for the seamless assembly of the editing fragment through Golden Gate cloning (63, 64) using AarI, a type IIS restriction enzyme that recognizes the sequence CACCTGC (4/8) and generates 4-bp overhangs outside its recognition site. To allow for phenotypic colony selection of the clones carrying the successfully assembled editing fragment by the naked eye, the AarI recognition sites were positioned to flank a codon-optimized *mCherry* variant (39) in the pLDEF Vector. In addition, pLDEF Helper provides the P*_ldh_*-*epheS*-*ermR* selection module, also flanked by two AarI sites. As depicted in Fig. 3B, the two standardized vectors can be combined in a Golden Gate cloning reaction with PCR-amplified, locus-specific DNA fragments corresponding to the homologous flanking regions and the direct repeat sequence necessary for latter excision of the selection module, resulting in the assembly of an editing fragment for gene deletion. Upon assembly of the editing fragment, *mCherry* is eliminated, resulting in white, nonfluorescent colonies of the desired transformants. For genomic insertions, the necessary insert sequence can be incorporated into the editing fragment during assembly in the form of an additional PCR product with flanking AarI sites, or it can be derived from a plasmid vector, if flanked by properly oriented AarI sites, such as the case of the *tetR*-P*_xyl/2tetO_* cassette in vector pLABID-mC and derivatives (Fig. 1A). In both cases, pLDEF Vector variants carrying the assembled editing fragments are selected in the presence of chloramphenicol, which eliminates background colonies formed by any undigested pLDEF Helper that carries the erythromycin resistance gene from the P*_ldh_*-*epheS*-*ermR* cassette. Colonies containing the undigested pLDEF Vector, on the other hand, can be easily spotted due to the expression of mCherry, which results in fluorescent and visibly pink colonies. As an additional feature, we included 8-bp-long recognition sites of the rare cutter NotI flanking the assembly area in pLDEF Vector, allowing for release of the assembled editing fragment through plasmid restriction digestion (Fig. 3C). After preparation, the linear DNA editing fragment can be directly introduced to S. thermophilus via natural competence, where it integrates to the chromosomal target locus via homologous recombination, without introducing unwanted nucleotide base pairs from the flanking scars generated during NotI digestion (Fig. 3D). The absence of the flanking scars in the edited genome is attributed either to the molecular mechanism of double-crossover homologous recombination, as the scars are located outside the two homologous regions of the editing fragment, or to the absence of homology between them and the genome.

Golden Gate assembly requires all used sequences to be domesticated by eliminating any internal restriction binding sites of the selected type IIS enzyme to avoid unintentional digestion (65). For the LDEF tool, using undomesticated sequences is unavoidable, as the homologous flanking and direct repeat regions are target specific and thus vary in sequence composition. We selected AarI as the type IIS restriction enzyme of choice, because its 7-bp-long recognition sequence renders it a rarer cutter than the most commonly used BsaI, BsmBI, and BbsI enzymes, reducing the cleavage frequency by up to 50% in the genome of S. thermophilus ST6. In addition, the 4-bp-long overhangs generated by AarI allow for higher assembly fidelity for multicomponent cassettes, compared to SapI, another 7-bp rare cutter used for Golden Gate assembly generating only 3 bp overhangs. Despite selecting rare cutters for our design, encountering AarI or NotI sites in any of the assembling parts cannot always be avoided. In such rare cases, strategically placed base alterations could eliminate any interfering recognition sites.

Alternative approaches for the assembly of linear DNA fragments, such as overlap extension PCR (OE-PCR) (66) or Gibson assembly (67) optimally require the exclusion of sequences containing tandem repeats or homopolymers, or that are prone to secondary structure formation, as this lowers the overall assembly efficiency. On the contrary, Golden Gate assembly is not subject to these limitations, allowing multicomponent assembly in one step, as long as internal restriction sites of the type IIS restriction enzymes being used are eliminated. This is highly relevant in the case of the LDEF tool presented here, as it is used for the assembly of linear DNA editing fragments that contain two 300-bp-long direct repeats. These repeats are necessary for selection marker excision during mutagenesis and thus cannot be avoided, but they may inhibit proper assembly when technologies relying on extensive complementation of single-stranded DNA ends are used, such as OE-PCR or Gibson assembly (53).

### *mCherry* induction by improved P*_tetR_* variants is dose- and time-dependent in both L. lactis and S. thermophilus.

After obtaining ATC-responsive variants of pLABID-mC in L. lactis MG1363 and genomic integrands of S. thermophilus ST6 expressing the *tetR*-P*_xyl/2tetO_*-*mCherry* cassette, we further characterized this inducible system in both organisms.

**FIG 4:**
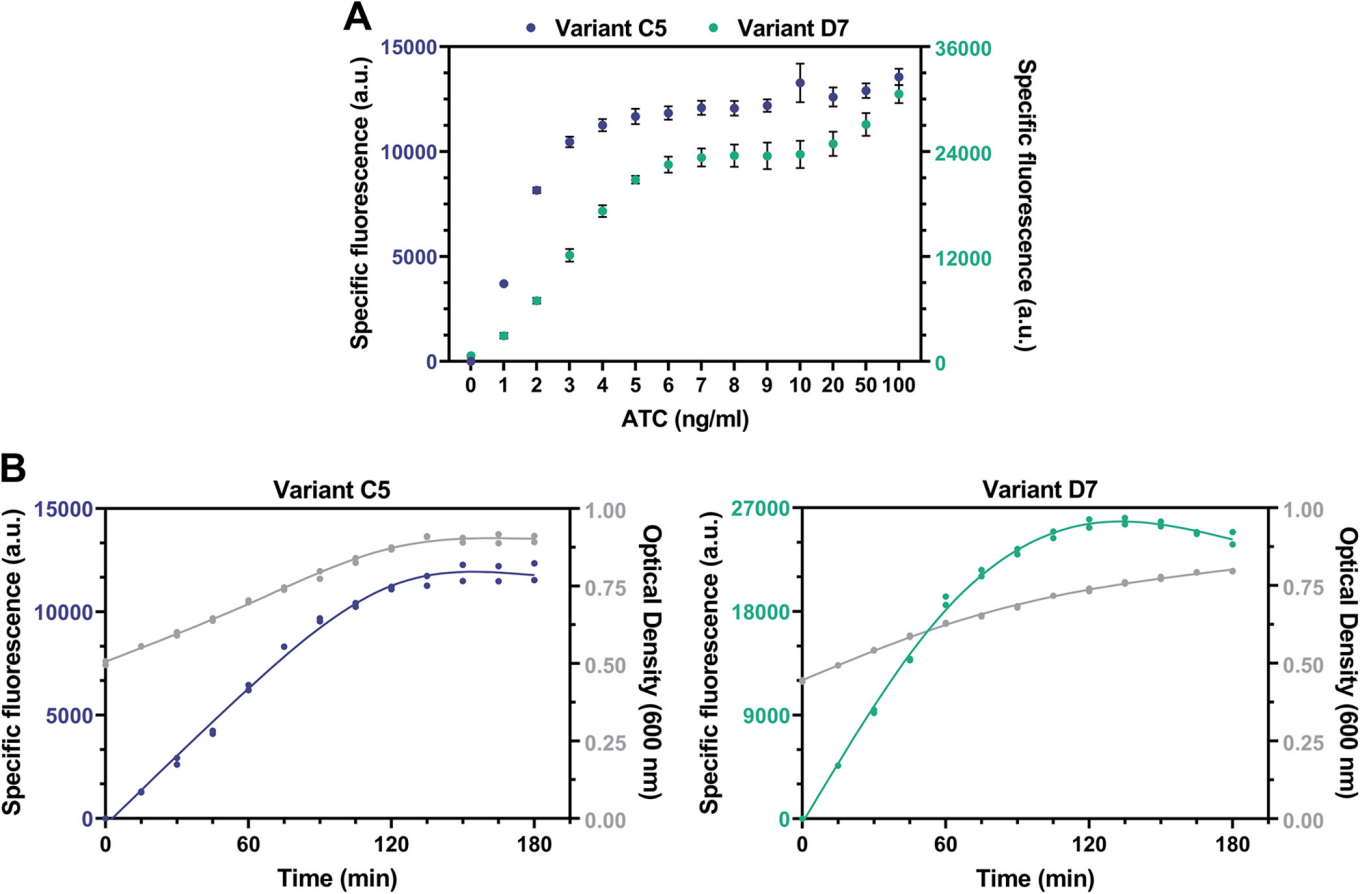
Characterization of pLABID-mC variants C5 and D7 in L. lactis. (A) ATC dose-dependent activation of P*_xyl/2tetO_* from pLABID-mC variants C5 and D7 in L. lactis MG1363, as indicated by expression of the fluorescent mCherry. Specific fluorescence was measured in cells cultivated overnight after the addition of different concentrations of ATC. (B) Time course of P*_xyl/2tetO_* promoter induction in pLABID-mC variants C5 and D7 in L. lactis MG1363. Cells in exponential growth were induced with 100 ng mL^−1^ ATC and quenched with 100 μg mL^−1^ chloramphenicol and erythromycin at 15-min intervals to inhibit protein synthesis.

From the obtained P*_tetR_* promoter variants of pLABID-mC in L. lactis (Fig. 2), two were selected for further characterization. Variant pLABID-mC-C5 exhibited the highest fluorescence signal in the presence of ATC without any detectable basal expression, which makes it especially suitable for applications that require tight transcriptional control. On the other hand, variant pLABID-mC-D7 showed one of the highest mCherry levels in the induced state, while retaining a relatively low basal expression, which could be more suitable for applications requiring high protein yield rather than tight control. Both variants proved highly responsive to ATC, with noticeable fluorescence observed using as little as 1 ng mL^−1^ of ATC (Fig. 4A). Gradual induction was achievable using higher ATC concentrations, with a linear response between fluorescence signal and ATC concentration in the ranges of 0 to 3 ng mL^−1^ and 0 to 5 ng mL^−1^ ATC for variants C5 and D7, respectively. Both variants reached full induction in the presence of 100 ng mL^−1^ of ATC, with higher concentrations having an impact on cell growth (Fig. S1).

Induction of exponentially growing cells using 100 ng mL^−1^ ATC, followed by subsequent quenching in regular intervals with chloramphenicol and erythromycin to stall protein synthesis and allow for mCherry maturation, provided us with the time course of induction. Both variants showed a detectable level of fluorescence in the first 15 min after induction, indicative of a fast response, which was 3.5-fold higher for variant D7 than for C5 (Fig. 4B). In agreement with the less strict control of P*_xyl/2tetO_* by TetR levels in variant D7, the fluorescence level increased with a higher rate for variant D7 than C5. Predictably, mCherry levels plateaued as cells entered stationary phase.

**FIG 5:**
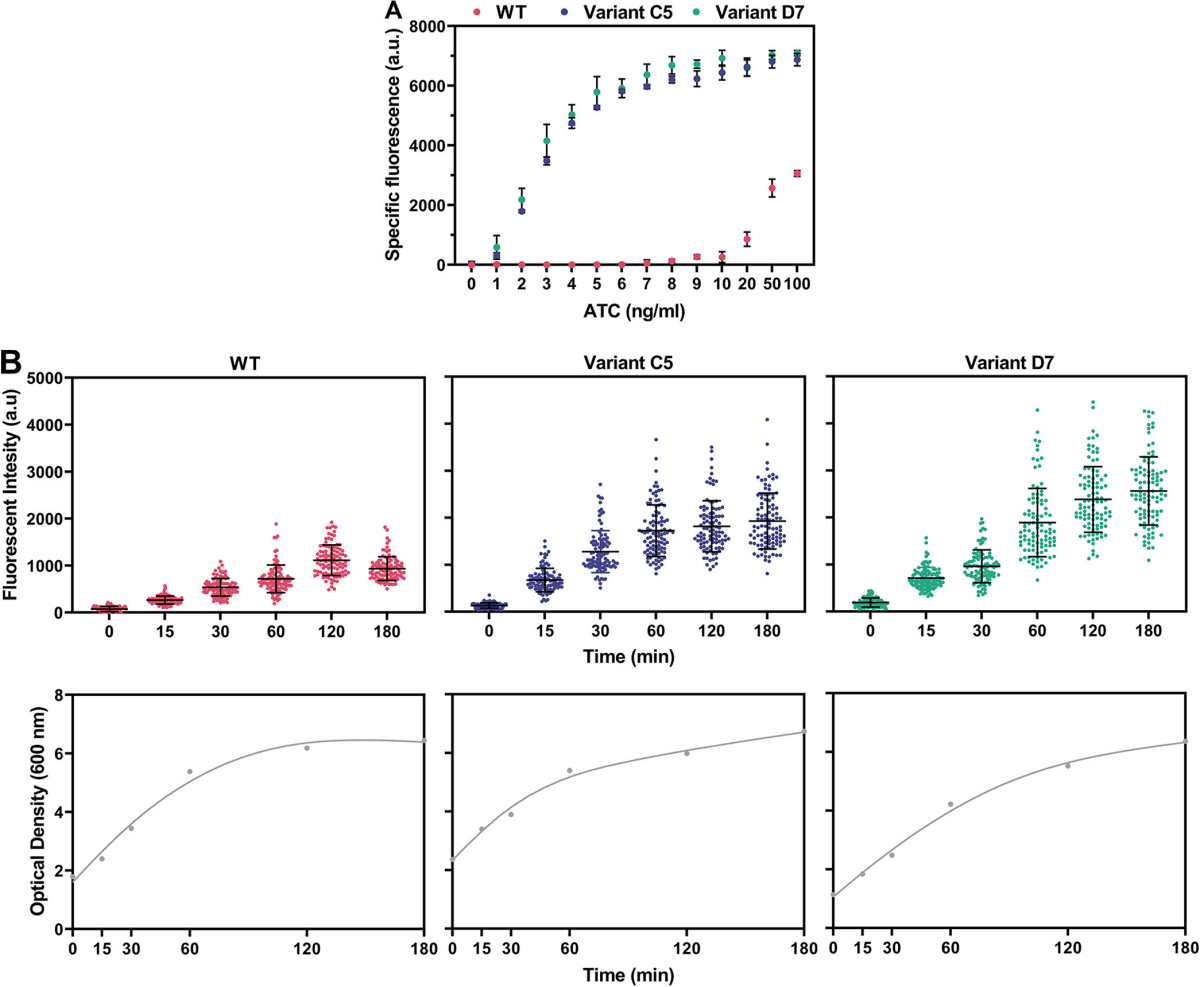
Characterization of S. thermophilus strains chromosomally expressing the *tetR*-P*_xyl/2tetO_*-*mCherry* cassette and its variants, C5 and D7. (A) ATC dose-dependent activation of P*_xyl/2tetO_* from cassettes expressing the wild-type P*_tetR_* promoter, or variants C5 and D7, in S. thermophilus ST6, as indicated by expression of mCherry. (B) Time course of P*_xyl/2tetO_* promoter induction in three derivatives of S. thermophilus ST6 expressing different variants of the P*_tetR_* promoter. In the top plots, each dot represents the fluorescence intensity of a single cell, with ~100 cells analyzed for each time point and strain.

In the *tetR*-P*_xyl/2tetO_*-*mCherry* genomic integrands of S. thermophilus ST6, P*_tetR_* promoter variants C5 and D7 showed a similar dose response, with linear mCherry expression observed for both in the ATC range of 0 to 5 ng mL^−1^ (Fig. 5). Interestingly, the WT *tetR*-P*_xyl/2tetO_*-*mCherry* cassette was also ATC responsive upon genomic integration in S. thermophilus, although higher concentrations of ATC, starting from 10 ng mL^−1^, were required for mCherry induction, with a less steep dose response and smaller dynamic range of induction being observed (Fig. 5A). The difference in behavior of the WT P*_tetR_* variant in S. thermophilus compared to L. lactis, where no mCherry expression was observed after full induction (Fig. 1B), could be the result of variations in copy numbers. The *tetR*-P*_xyl/2tetO_*-*mCherry* cassette is present in a single copy in S. thermophilus, while it is expressed from multiple plasmids in L. lactis, likely resulting in different intracellular levels of TetR and, consequently, dissimilar responses under the same induction conditions.

To observe the time course of induction in S. thermophilus mutants expressing the *tetR*-P*_xyl/2tetO_*-*mCherry* cassette, we measured single-cell fluorescence in samples derived from cultures induced with 100 ng mL^−1^ ATC during exponential phase (Fig. 5B). All variants showed increasing levels of fluorescence after 15 min of induction, with the WT variant exhibiting the slowest response over time, presumably as a result of higher TetR expression. Variants C5 and D7 showed similar responses (Fig. 5B), although both the fluorescent intensity and course of induction were lower and slower, respectively, than what was observed for these variants in L. lactis (Fig. 4). This highlights yet again the effect that different copy numbers and hosts can have on the performance of the same genetic module. These results together depict a sensitive and fast tetracycline-inducible system, functional in two industrially important lactic acid bacteria.

### Conclusions.

In this study, we adapted the popular TetR-controlled expression system for the lactic acid bacteria L. lactis and S. thermophilus using the *tetR*-P*_xyl/2tetO_* module from the staphylococcal vector pRAB11. Despite the proven functionality of the TetR-controlled promoter system in other Gram-positive bacteria, such as S. aureus (35) and S. pneumoniae (36), the system was constitutively repressed in L. lactis. By optimizing the promoter and RBS sequence of the tetracycline repressor TetR, ATC-controlled expression of the reporter gene coding for mCherry was achieved in both L. lactis and S. thermophilus. This observation illustrates how the functionality of genetic elements is heavily dependent upon the expression host and highlights the need for optimization of synthetic biology tools to new genetic backgrounds.

The popularity of tetracycline-controlled expression systems relies in part on the simplicity of induction. Anhydrotetracycline, a derivative of tetracycline with reduced antibacterial activity, enters cells through diffusion and can be used in different growth media. In contrast, systems that use sugars, metals, or peptides as the inducing molecules can have several disadvantages. Induction via sugars requires as a prerequisite the presence of the necessary membrane transporters for sugar uptake, which can be limiting for some bacteria. S. thermophilus, for example, has undergone extensive regressive evolution by adapting to its niche environment, milk (68). This has led to a very specific sugar uptake profile due to loss of function in several sugar transporters, which inhibits the use of many sugar-dependent expression systems. Sugars can also interfere with carbon metabolism, considerably affecting bacterial growth due to the carbon metabolism repression response, which regulates the sequential utilization of carbohydrates (69). During induction using metal ions, medium composition must be carefully controlled to eliminate intrinsic levels of the inducer, as this can lead to high basal expression levels. This often demands the use of chemically defined media or the addition of chelating agents. Moreover, inducing peptides can be expensive due to their synthesis cost, as well as sensitive to proteolysis. Use of anhydrotetracycline is not subject to these limitations, and as demonstrated in this study, a linear dose response can be achieved using concentrations well below the limit where bacterial growth is inhibited.

In addition, we implemented a previously described markerless mutagenesis method (53) in S. thermophilus, and to improve upon its ease of use, we developed a set of vectors for efficient assembly of the relevant linear DNA editing fragments using Golden Gate assembly (63, 64). This method relies on the transformation of only a single linear DNA editing fragment expressing an antibiotic resistance gene and the counterselection gene *epheS*, both flanked by direct repeat sequences that eventually allow for marker excision through intramolecular recombination after genomic integration (Fig. 3A). Compared to previously described mutagenesis methods for S. thermophilus, this approach does not require the heterologous expression of any enzymes, such as the Cre recombinase (32), This eliminates the need for plasmid curing and minimizes plasmid compatibility issues, as the transformation efficiency of a single plasmid can vary tremendously between industrial strains of S. thermophilus based on our personal experience. In addition, only a single transformation step is needed for the present method, unlike other approaches (34), where multiple integration events are required, making them more time-consuming and unfit for targeting genes involved in natural competence development or DNA recombination. A drawback of all the aforementioned methodologies is the dependence on natural competence for the transformation of the linear DNA editing fragments, which consequently restricts their application to naturally transformable strains only.

The inducible promoter and mutagenesis method described here enriches the molecular toolbox of two key LAB that are extensive used in the fermented food industry. Microbes generated by such means are classified as genetically modified organisms (GMOs) under current legislation. This prohibits their application in any relevant food applications in the European Union, although regulations appear more permissive in other countries (70, 71). Nonetheless, genetic engineering of industrially relevant strains can advance our understanding of complex cellular mechanisms and traits, which can eventually provide insights for the generation of enhanced strains by natural improvement strategies.

## MATERIALS AND METHODS

### Bacterial strains and growth conditions.

The bacterial strains used in this study are described in Table 1. Escherichia coli strain DH5α (New England Biolabs) was used for propagation of plasmid pSMQ001 and grown in LB broth (72) supplemented with 100 μg mL^−1^ ampicillin at 37°C under constant agitation. Lactococcus lactis strain MG1363 (73) was used for all cloning purposes and routinely grown in M17 broth (BD Difco) (74) containing 0.5% (wt/vol) glucose (GM17), without agitation at 30°C, unless otherwise stated. The broth was supplemented with 5 μg mL^−1^ of chloramphenicol or 5 μg mL^−1^ of erythromycin, when required. Streptococcus thermophilus strains were grown in M17 broth containing 2% (wt/vol) lactose (LM17), without agitation at 40°C and under anaerobic conditions (9 to 13% CO_2_) created using gas-generating sachets (Thermo Scientific Oxoid AnaeroGen), unless otherwise stated. For antibiotic resistance selection, 10 μg mL^−1^ chloramphenicol or 1 μg mL^−1^ erythromycin were added to the culture broth. For maximum P*_xyl/2tetO_* promoter induction, 100 ng mL^−1^ anhydrotetracycline (ATC) (Cayman Chemical) was used in both species.

**TABLE 1:** Strains and plasmids used in this study

Strain or plasmid	Characteristics^*a*^	Source or reference
Strains		
E. coli DH5α	*fhuA2* Δ(*argF*-*lacZ*)U169 *phoA glnV44* ϕ80Δ(*lacZ*)M15 *gyrA96 recA1 relA1 endA1 thi-1 hsdR17*	New England Biolabs
L. lactis MG1363	Plasmid-free and prophage-cured derivative of dairy strain NCDO712	MoBiTec (73)
S. thermophilus		
ST6	Texturizing dairy strain; Chr. Hansen A/S culture collection	Chr. Hansen A/S
ST6-mC	ST6 derivative with *tetR*-P*_xyl/2tetO_*-*mCherry* insertion	This study
ST6-mC-C5	ST6 derivative with optimized *tetR*-P*_xyl/2tetO_*-*mCherry* insertion	This study
ST6-mC-D7	ST6 derivative with optimized *tetR*-P*_xyl/2tetO_*-*mCherry* insertion	This study
Plasmids		
pEFB001	pNZ8148 derivative with P*_11_*-*mCherry*; Cm^r^	39
pIL252	pAMβ1 derivative, broad-host-range; Erm^r^	37
pIL252CmR	pIl252 derivative with *cat* from pNZ8148; Cm^r^	Chr. Hansen A/S
pLABID	pIL252CmR derivative with *tetR* and P*_xyl/2tetO_* of pRAB11; Cm^r^	This study
pLABID-mC	pLABID derivative with *mCherry* of pEFB001 under P*_xyl/2tetO_*; Cm^r^	This study
pLABID-mC-C5	pLABID-mC variant with optimized *tetR* expression; Cm^r^	This study
pLABID-mC-C5-ErmR	pLABID-mC-C5 derivative; Erm^r^	This study
pLABID-mC-D7	pLABID-mC variant with optimized *tetR* expression; Cm^r^	This study
pLABID-mC-D7-ErmR	pLABID-mC-D7 derivative; Erm^r^	This study
pLABID-mC-ErmR	pLABID-mC derivative; Erm^r^	This study
pLABID-mC-ΔtetR	pLABID derivative without *tetR*; Cm^r^	This study
pLDEF Helper	pNZ8148 derivative with P*_ldh_*-*epheS*-*ermR* of pSMQ001 flanked by AarI sites; Erm^r^	This study
pLDEF Vector	pNZ8148 derivative with P*_11_*-*mCherry* of pEFB001 flanked by AarI and NotI sites; Cm^r^	This study
pLDEF-tetR-mC	pLDEF Vector derivative with *tetR* and P*_xyl/2tetO_*-*mCherry* of pLABID-mC and P*_ldh_*-*epheS*-*ermR* from pLDEF Helper; Cm^r^ Erm^r^	This study
pLDEF-tetR-mC-C5	pLDEF Vector derivative with *tetR* and P*_xyl/2tetO_*-*mCherry* of pLABID-mC-C5 and P*_ldh_*-*epheS*-*ermR* from pLDEF Helper; Cm^r^ Erm^r^	This study
pLDEF-tetR-mC-D7	pLDEF Vector derivative with *tetR* and P*_xyl/2tetO_*-*mCherry* of pLABID-mC-D7 and P*_ldh_*-*epheS*-*ermR* from pLDEF Helper; Cm^r^ Erm^r^	This study
pNZ8148	pSH71 derivative with nisin A promoter (P*_nisA_*), broad-host-range vector; Cm^r^	62
pRAB11	pRMC2 derivative with *tetR* and P*_xyl/2tetO_*; Amp^r^ Cm^r^	35
pSMQ001	pUC57 derivative with P*_ldh_*-*epheS*-*ermR*; Amp^r^ Erm^r^	Chr. Hansen A/S

a Cm^r^, chloramphenicol resistance; Erm^r^, erythromycin resistance; Amp^r^, ampicillin resistance.

### Plasmid vector generation.

All standard molecular techniques were performed according to reference 72. The plasmids and primers used in this study are listed in Table 1 and Table 2, respectively. All PCR amplifications were carried out using the high-fidelity Q5 DNA polymerase from New England Biolabs in the form of a 2× master mix, following the manufacture’s routine protocol for setting up the reactions and thermocycling conditions. For construction of pLABID, two synthetic DNA fragments, one containing the *tetR* gene under its respective promoter and part of the promoter P*_xyl/2tetO_* from pRAB11 (35), and the other the remaining operator site of P*_xyl/2tetO_* together with the multiple-cloning site of pRAB11, were gene synthesized (Twist Biosciences). Both fragments were designed to contain AarI restriction sites flanking the *tetR*-P*_xyl/2tetO_*-GOI (gene of interest) region to allow for compatibility of the generated vector with the LDEF Assembly tool described above, as well as flanking BsaI recognition sites for Golden Gate cloning-based assembly of the vector (63, 64). The two synthesized fragments were combined with the backbone of pIL252CmR, a derivative of pIL252 (37), amplified using primers P341 and P342, and treated with BsaI-HFv2 and T4 DNA ligase. Subsequently, the assembly reaction was purified and electroporated into L. lactis MG1363 (75). Constructs were verified by Sanger sequencing and plasmid DNA was isolated using the Nucleospin plasmid kit (Macherey-Nagel), after pretreatment of the cells in lysis buffer (20% [wt/vol] sucrose, 10 mM Tris-HCl [pH 8.1], 10 mM EDTA, 20 mM NaCl, 30 μg mL^−1^ RNase A, 10 mg mL^−1^ lysozyme) for 30 min at 55°C.

**TABLE 2:** Oligonucleotides used in this study

Name	Sequence (5′→3′)^*a*^
P132	AGATGAGGACAGCAGGTGACCAAGGCTTGAAACGT
P133	TAGTCTGCAGCAGGTGTGTTGAACTAATGGGTGCT
P134	GCCTTGGTCACCTGCTGTC**CTCA**TCTGGATTTACC
P135	AGTTCAACACACCTGCTGCA**GACT**AGTTTGTACCA
P144	CGGCCGCCATCGCAGCAGGTGAGCGCTATAGTTGTTGACA
P145	CGGCCGCTGCTTAAGCAGGTGGCTACGATAACGCCTGT
P146	CGTAGCCACCTGCTTAA**GCA*G****CGGCCGC*GGCGTTATCGTAGCGTA
P147	AGCGCTCACCTGCTGCG**ATG*G****CGGCCGC*GGGCAGGTTAGTGACA
P341	GGCTACGGTCTCCTGATCCGTAGCGGTTTTCAAAATTTG
P342	GGCTACGGTCTCACTTCGCTCTCACTGCCCC
P365	GATAGAGTATGATGGAGGAGGTTTCTAGAGTGAGTAAAGG
P366	GCTTTTATTTGTACAGCTCATCCATGC
P367	GCATGGATGAGCTGTACAAATAAAAGCCAGCCAAGCTTATCGATTCTAGAC
P368	CTCACTCTAGAAACCTCCTCCATCATACTCTATCAATGATAGAGAGC
P370	GGCTACCACCTGCCGCG**CCAT**CTGCTAGGACGCTTTGTG
P371	GGCTACCACCTGCATTC**CGTC**GAGTTGGCAATCCACATAC
P372	GGCTACCACCTGCATAT**ACTC**GGAACAAGCTCAGCAAGC
P373	GGCTACCACCTGCGGCG**AGTC**GGAACAAGCTCAGCAAGC
P374	GGCTACCACCTGCCAGC**TGAG**GGAACAAGCTCAGCAAGC
P375	GGCTACCACCTGCATTT**CTGC**CCGTTTCTACAGCTGCCG
P401	GATCGCCTCGAGTTCATG
P402	GATACCGTCTCAAGCAGGTG
P403	GTTGACATTATATCATTGATAGAG
P404	GATTAACAGCGCATTAGAG
P405	CCGACCTCATTAAGCAGCTCTAATGCGCTGTTAATCACTTTACTTTTATCTAATCTAGACATCATTAATT**NNNCCT**TTTTGTTGACATTATATCATTGATAGAGTTATTTGTCAAACTAG
P406	CCGACCTCATTAAGCAGCTCTAATGCGCTGTTAATCACTTTACTTTTATCTAATCTAGACATCATTAATT**CCTNNN**TTTTGTTGACATTATATCATTGATAGAGTTATTTGTCAAACTAG
P407	CCGACCTCATTAAGCAGCTCTAATGCGCTGTTAATCACTTTACTTTTATCTAATCTAGACATCATTAATT**NNNNNN**TTTTGTTGACATTATATCATTGATAGAGTTATTTGTCAAACTAG
P586	AAGAGTGTGTTGATAGTGCAGTATCTTAA
P587	ATAGAATTATTTCCTCCCGTTAAATAATAGATAAC
P588	ATTATTTAACGGGAGGAAATAATTCTATGAGTCGC
P589	GATACTGCACTATCAACACACTCTTAAGTTTGC

a Underlining indicates AarI restriction sites used for cloning, boldface letters indicate the base pairs digested by AarI during cloning, and italic letters indicate NotI restriction sites used for linear DNA editing fragment release from pLDEF Vector.

For pLABID-mC, the low-GC-content codon-optimized variant of *mCherry* was amplified from pEFB001 (39) using primers P365 and P366, while the pLABID backbone was amplified using primers P367 and P368. The two DNA fragments were ligated using the NEBuilder HiFi DNA assembly reagent (New England Biolabs). For construction of pLABID-mC-ΔtetR, primers P401 and P402 were phosphorylated by T4 polynucleotide kinase and used to amplify the whole pLABID-mC excluding the *tetR* gene. After an additional DpnI treatment, the DNA fragment was purified and ligated using T4 DNA ligase. For both vectors, cloning resumed as described above with electroporation in L. lactis.

For construction of the pLDEF Vector plasmid, primers P114 and P145 were used for amplification of *mCherry* from pEFB001 together with the strong constitutive synthetic promoter P*_11_* of Lactobacillus plantarum (76), and primers P146 and P147 for the backbone of plasmid pNZ8148 (MoBiTec). NotI and AarI sites flanking the *mCherry* gene were introduced to the construct by primer overhangs. For the pLDEF Helper plasmid, primers P132 and P133 were used for amplification of the pNZ8148 backbone without the chloramphenicol resistance gene, while primers P134 and P135 for the amplification of the P*_ldh_*-*epheS*-*emrR* cassette from plasmid pSMQ001. Both plasmids were constructed using the NEBuilder HiFi DNA assembly reagent, with cloning resumed as described above.

To exchange the chloramphenicol resistance gene to that of erythromycin in pLABID-mC and its derivatives, primers P586 and P587 were used for amplification of the *emrR* gene from pIL252 (37), while primers P588 and P589 were used for the amplification of the pLABID-mC backbone and that of its RBS-optimized variants pLABID-mC-C5 and pLABID-mC-D7. The vectors were ligated by the NEBuilder HiFi DNA assembly reagent. Cloning resumed as described above, and the isolated plasmids were transformed to S. thermophilus ST6 as described in reference 77, using a modified electroporation buffer (0.5 M sucrose, 10% [wt/vol] glycerol) and by delivering two consecutive electric pulses to the cell suspension.

### Screening for ATC-responsive pLABID mutant variants.

For disruption of the *tetR* ribosomal binding site sequence in pLABID-mC, three degenerate oligonucleotides containing this region were synthesized (P405–P407), pooled, and mixed with the backbone of pLABID-mC, which was amplified using primers P403 and P404. The vector library was constructed by single-strand DNA oligonucleotide assembly using the NEBuilder HiFi DNA reagent. Cloning was resumed as described above for L. lactis MG1363. The resulting vector library was screened for optimal TetR expression level by selecting individual colonies and plating them on GM17 agar plates containing 5 μg mL^−1^ chloramphenicol, supplemented or not with 500 ng mL^−1^ ATC. Expression of mCherry was accessed by phenotypic observation of colonies under blue light using a BLooK LED Transilluminator (GeneDireX).

After this initial screening, fluorescence levels were determined for a selected number of clones. For this, cells were grown to stationary phase in GM17 containing 5 μg mL^−1^ chloramphenicol in 96-well plates sealed with an air-permeable membrane (Breathe-Easy; Diversified Biotech), in the presence or absence of 100 ng mL^−1^ ATC. Optical density at 600 nm (OD_600_) and fluorescence were determined using a Synergy H1 microplate reader (BioTek). mCherry fluorescence was measured with an excitation of 579 nm and emission of 616 nm, while the gain was set at 100. OD_600_ measurements were used to correct for mCherry fluorescence by calculating the specific fluorescence (fluorescence per OD value defined in arbitrary units). Plate assays were performed in three biological replicates with technical quadruplicates.

### Dose-response assays.

To characterize the induction capabilities of pLABID-mC-C5 and pLABID-mC-D7 under different concentrations of ATC in L. lactis, cells were grown to stationary phase in GM17 containing 5 μg mL^−1^ chloramphenicol and various concentrations of the inducer (0 to 100 ng mL^−1^ ATC) in 96-well plates sealed with an air-permeable membrane. The specific fluorescence was determined as described above after 24 h of aerobic incubation at 30°C, a time point selected for its higher fluorescence levels after a preliminary screening of different maturation times (16 to 48 h).

For S. thermophilus strains with insertion of the *tetR*-P*_xyl/2tetO_*-*mCherry* cassette, the dynamic range of mCherry induction was estimated in cells grown to stationary phase in LM17 containing various concentration of the inducer (0 to 100 ng mL^−1^ ATC) in 96-well plates. After overnight incubation at 40°C under anaerobic conditions, generated as described above, cells were quenched with 200 μg mL^−1^ chloramphenicol and erythromycin to stop further protein synthesis. Specific fluorescence was determined as described above after incubating the quenched cells aerobically for 24 h at room temperature to allow for complete mCherry maturation.

### Time-response assays.

To determine the time response of induction in L. lactis, cells were grown to an OD_600_ of 1, induced with 100 ng mL^−1^ ATC, and aliquoted in a 96-well plate. Incubation at 30°C continued using a Thermomixer C instrument (Eppendorf) equipped with a 96-well plate thermoblock, and cells were quenched in each time point with 200 μg mL^−1^ chloramphenicol and erythromycin. The plates were sealed with an air-permeable membrane and incubated for 24 h at room temperature in the dark to allow for mCherry maturation. Specific fluorescence was determined as described above using a microplate reader. All experiments were performed in biological duplicates with technical quadruplicates.

To study the time response of mCherry induction in S. thermophilus derivatives expressing the *tetR*-P*_xyl/2tetO_*-*mCherry* cassette chromosomally, cells were grown overnight in crimp top serum bottles containing LM17 flushed with N_2_, under constant agitation (400 rpm) at 40°C. The next day, fresh cultures were inoculated to an initial OD_600_ of 0.1 under the same growth conditions. When the OD_600_ reached ~1 to 2 (early to mid-exponential growth phase), cells were induced with 100 ng mL^−1^ ATC. Cell aliquots of 200 μL were retrieved at regular time intervals, placed in a 96-well plate, and quenched using 200 μg mL^−1^ chloramphenicol and erythromycin. The plate was sealed with an air-permeable membrane and treated as described above. For cell visualization, 1 μL of the bacterial suspension was deposited on a 1.5% agarose–phosphate-buffered saline (PBS) pad cast on top of a glass slide and covered with a coverslip. The slides were viewed using a Nikon Eclipse Ti2-E inverted microscope with a 100× phase-contrast objective (Nikon Plan Apo λ 1.45, oil immersion) and photographed using a Primer BSI sCMOS camera (Teledyne Photometrics) and the NIS-Elements software (Nikon). For mCherry visualization, excitation at 580 nm and emission at 610 nm were used. Image analysis for quantification of the fluorescence intensity per cell was performed using the Fiji package (78).

### Linear DNA editing fragment preparation.

Assembly of the linear DNA editing fragment containing the *tetR*-P*_xyl/2tetO_*-*mCherry* cassette for genomic integration in S. thermophilus was done using the dual-vector LDEF tool, designed in this study and described in the Results and Discussion section above.

Chromosomal DNA was isolated from S. thermophilus ST6 using the NucleoSpin microbial DNA minikit (Macherey-Nagel). Using this DNA as the template, primers pairs P370-P371, P372-P373, and P374-P375 were used for amplification of the two homologous regions flanking the insertion site, as well as for the direct repeat DNA sequence, respectively, introducing unique AarI restriction sites in the process by primer overhangs. All DNA fragments were purified using the Monarch PCR & DNA cleanup kit (New England Biolabs). The complete DNA editing fragment was assembled in vector pLDEF-tetR-mC using Golden Gate Assembly with AarI, as described in reference 79, with some modifications. The reaction was carried out in 15 μL by mixing 50 ng each of the pLDEF Vector, pLDEF Helper, and pLABID-mC plasmids, together with 3× the molar amount of the homologous region DNA fragments and 10× the molar amount of the direct repeat DNA fragment. To this mixture were subsequently added 1.5 μL 1 mg mL^−1^ bovine serum albumin (BSA), 1.5 μL 10× T4 DNA ligase buffer, 1.5 μL AarI (ThermoFisher Scientific), 0.5 μL T4 DNA ligase, and 0.5 μL 25× oligonucleotide containing the recognition site of AarI, as provided together with AarI, for optimal enzyme digestion. The reaction mixture was incubated in a thermocycler for maximum of 10 consecutive cycles at 37°C for 50 min and 16°C for 10 min, followed by a final digestion step at 37°C for 30 min and enzyme denaturation at 65°C for 20 min. After purification using the Monarch PCR & DNA cleanup kit, the reaction mixture was electroporated into L. lactis MG1363 as described above. After isolation, the assembled vector pLDEF-tetR-mC was digested with NotI and the released linear DNA editing fragment was purified by agarose gel extraction using the Monarch DNA gel extraction kit (New England Biolabs).

Vectors pLDEF-tetR-mC-C5 and pLDEF-tetR-mC-D7 were assembled in a similar manner, using vectors pLABID-mC-C5 and pLABID-mC-D7, respectively, instead of pLABID-mC in the Golden Gate assembly.

### Genomic insertion in S. thermophilus by natural transformation.

For genomic integration of the linear DNA editing fragment, S. thermophilus was transformed by natural competence as described in reference 23, with some modifications. In detail, S. thermophilus was grown overnight in chemically defined medium (see Table S1 in the supplemental material) in crimp top serum bottles with a headspace flushed with a gas mixture of 80% N_2_ (vol/vol) and 20% CO_2_ (vol/vol) under agitation (400 rpm) at 40°C. This preculture was used to inoculate a new culture to an initial OD_600_ of 0.05 the next day. When cells reached an OD_600_ of ~0.2, 10 μΜ of the competence peptide ComS_17–24_ (LPYFAGCL; purity of >95%) (TAG Copenhagen) were added to 300-μL culture aliquots, together with 0.1 to 1 μg of the linear DNA editing fragment. The mixture was incubated at 40°C for 3 h without agitation aerobically, until the addition of 700 μL LM17 and subsequent incubation at 40°C for 30 min. Cells were plated on LM17 agar plates containing 1 μg mL^−1^ of erythromycin, followed by incubation under anaerobic conditions, generated as described above, at 40°C overnight. Genomic integration by double-crossover homologous recombination was verified by colony PCR. For excision of the *epheS* and *ermR* selection genes after integration, colonies were grown in 200 μL LM17 for 2 to 6 h at 40°C and then plated on LM17 agar plates containing 5 mM 4-chloro-dl-phenylalanine (PCPA). Successful intramolecular recombination for selection marker excision was verified by colony PCR and Sanger sequencing.
