# Supplementary material for: Development of a Tetracycline-Inducible System for Conditional Gene Expression in Lactococcus lactis and Streptococcus thermophilus
Source: Microbiol Spectr. 2023 May 16;11(3):e00668-23. doi: 10.1128/spectrum.00668-23 (PMC10269922; doi:10.1128/spectrum.00668-23)
Supplement: Supplemental file 1 — Supplemental material. Download spectrum.00668-23-s0001.docx, DOCX file, 0.9 MB [file spectrum.00668-23-s0001.docx]

**Supplementary Information**

**Composition of chemical defined medium (CDM) for *Streptococcus thermophilus***

CDM was prepared similarly to [1] , with minor modifications. All solutions (Table S1) were prepared individually in crimp top serum bottles under anaerobic conditions and sterilized by autoclavation or membrane filtration. CDM was prepared by mixing the appropriate volume of each solution in a 250 ml crimp top serum bottle previously flushed with a gas mixture of 80 % N_2_ (v/v) and 20 % CO_2_ (v/v).

**Supplementary Table S1. Composition of CDM component solutions**

| **Component** | **Concentration (g L ^-1^)** | **Component** | **Concentration (g L ^-1^)** |
| --- | --- | --- | --- |
| **Basal solution (1x)** |  | **Amino acid solution (26.3x)** |  |
| K_2_HPO_4_ | 2.5 | L-Alanine | 40 |
| KH_2_PO_4_ | 3 | L-Arginine | 40 |
| NaHCO_3_ | 0.42 | L-Asparagine·H_2_O | 45.2 |
| Sodium acetate | 1 | L-Aspartate | 40 |
| Ammonium citrate | 0.6 | L-Glutamate | 40 |
| MnSO_4_·H_2_O | 0.0028 | L-Glutamine | 40 |
| FeCl_2_·4H_2_O | 0.005 | Glycine | 40 |
| **Trace element solution (1000x)** |  | L-Histidine·H_2_O·HCL | 54 |
| ZnCl_2_ | 0.07 | L-Isoleucine | 40 |
| MnCl_2_·4H_2_O | 0.1 | L-Leucine | 40 |
| FeCl_2_·4H_2_O | 1.5 | L-Lysine | 40 |
| H_3_BO_3_ | 0.006 | L-Methionine | 40 |
| CoCl_2_·6H_2_O | 0.19 | L-Phenylalanine | 40 |
| CuCl_2_·2H_2_O | 0.002 | L-Proline | 40 |
| NiCl_2_·6H_2_O | 0.024 | L-Serine | 40 |
| **Sugar solution (10x)** |  | L-Threonine | 40 |
| Lactose·H_2_O | 200 | L-Tryptophane | 40 |
| **Metal solution 1 (100x)** |  | L-Tyrosine | 40 |
| MgCl_2_·6H_2_O | 20 | L-Valine | 40 |
| **Metal solution 2 (100x)** |  | **Vitamin Solution (100x)** |  |
| CaCl_2_·2H_2_O | 5 | Biotin | 0.02 |
| **Urea solution (100x)** |  | Folic acid | 0.02 |
| Urea | 12 | Pyridoxal·HCl | 0.1 |
| **Base solution (100x)** |  | Riboflavin | 0.05 |
| Adenine | 1 | Thiamine·HCl | 0.05 |
| Guanine | 1 | Nicotinamide | 0.05 |
| Uracil | 1 | Cobalamin | 0.05 |
| Xanthine | 1 | p-Aminobenzoic acid | 0.05 |
| **Cysteine solution (100x)** |  | DL-Ca-Pantothenate | 0.4 |
| Cysteine·HCl | 50 | DL-6,8-Thioctic acid | 0.5 |


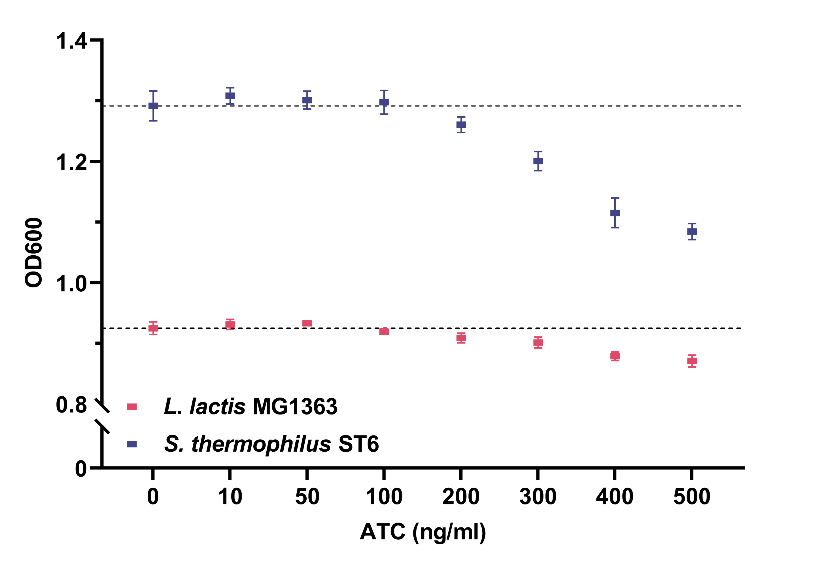


**Supplementary Figure S1. Dose response of L. lactis MG1363 and S. thermophilus ST6 to anhydrotetracycline (ATC).** Cells from overnight cultures were inoculated in LM17 (for S. thermophilus) or GM17 (for L. lactis) containing different concentrations of ATC in a 1/40 ratio. For S. thermophilus, the inoculated 96-well plate was incubated overnight at 40 °C anaerobically, while for L. lactis the plate was sealed with a not air-permeable film and incubated at 30 °C. The next day the optical density at 600 nm was measured using a microplate reader. Concentration of ATC greater than 100 ng ml^-1^ led to growth inhibition for both microorganisms.


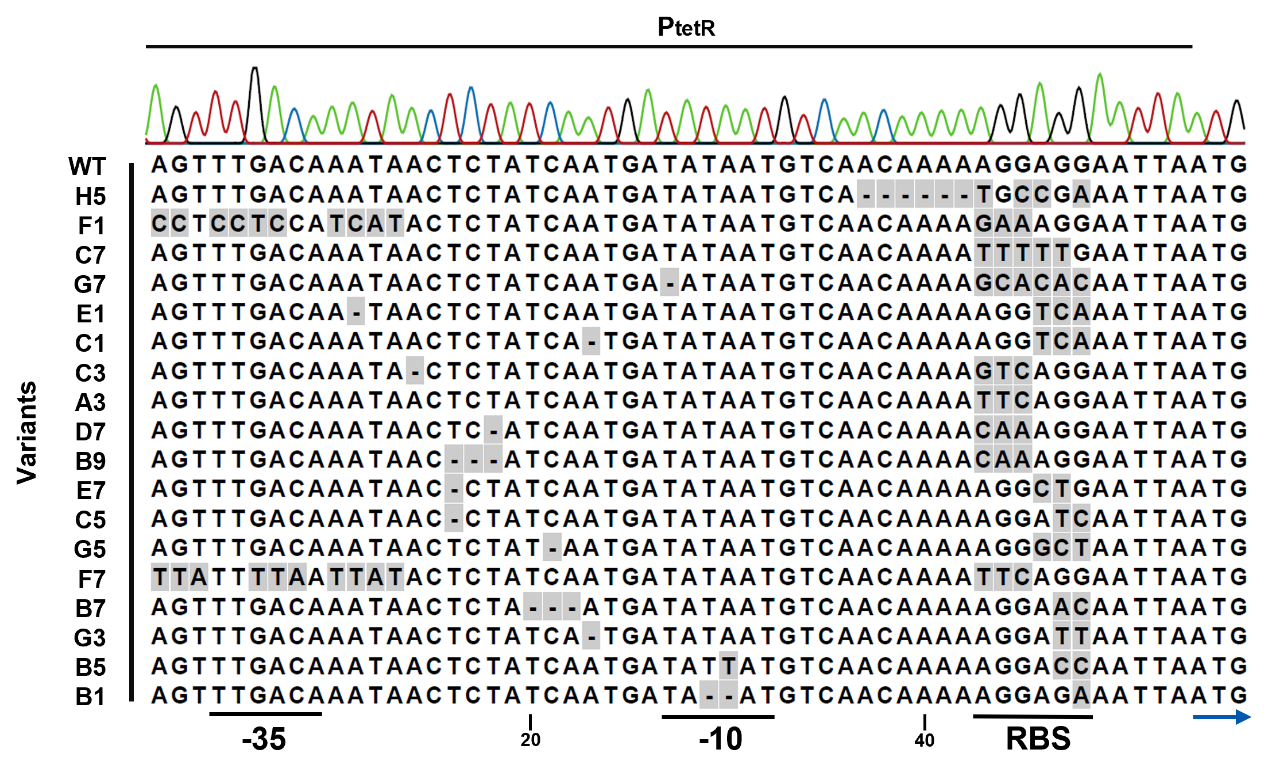


**Supplementary Figure S2.** **Mutation analysis of all selected pLABID-mC variants after library screening.** Letters or dash lines marked with grey represent deviations from the original sequence of the promoter P_tetR_, indicated here as WT (wild type). Blue arrow indicates the starting codon of tetR.


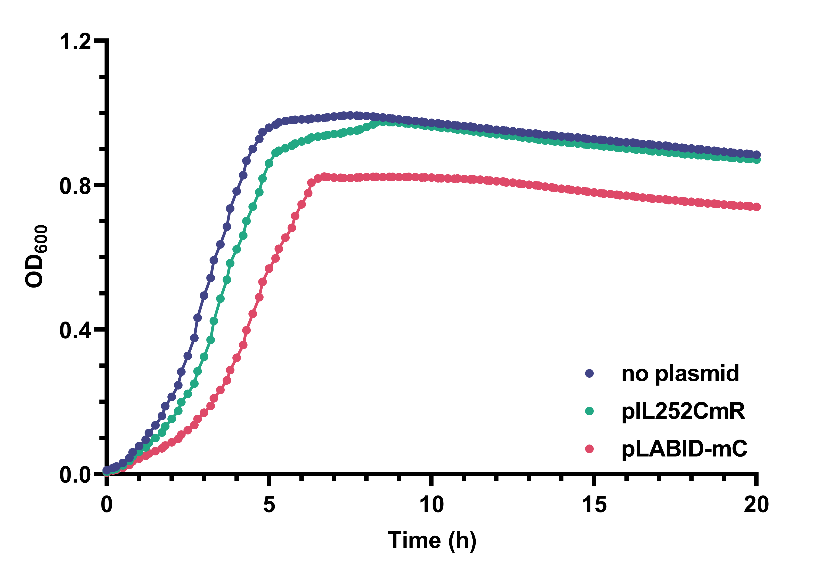


**Supplementary Figure S3. Comparative growth of L. lactis MG1363 in complex medium.** Cultures grown overnight in M17 medium supplement with 0.5 % glucose at 30 °C were adjusted to OD_600_=1 and then used in a ratio of 1/40 for the inoculation of a 96-well plate. Chloramphenicol was added in a final concentration of 5 μg ml^-1^, when needed. The plate was sealed with an air permeable membrane and incubated at 30 °C using a Synergy H1 microplate reader, with the OD_600_ measured every 10 min for 20 h. The depicted lines are the average of three biological replicates.

ATGGATTTACAAACACAATTACAAGAGTTGAAAACCTCAACGCA**A**GC**A**AAA**C**T**T**GC**A**GAAATGCGTGGTGAACACTCTAAGGAATTACAAGAATTGCGTGTTGCAGTTTTAGGTAAAAAAGGTTCTTTGACGGAA**T**T**GC**T**T**AA**A**GG**T**CTTAAAGACCTTCCTAGTGAAGAACGCCCTACAGTTGGTAAAATGGTCAACGAAGTTCGTGACGTT**C**T**T**AC**A**GAAGC**A**TTTGACGAAGCAGCTAAAGTTGTTGAAGCAGCTAAAATTCAAGCCCAACTTGACTCTGAAAGTCTTGA**T**GT**T**AC**TT**T**G**CCAGGTCGCCAAGTAAACCTTGGTAATCGTCATATTCTTAGCCAAATAGCTGAAGAAATCGAAGATATTTT**TC**T**T**GG**T**ATGGG**T**TTCCAAATTGTTGATGGTTTTGAAGTTGAAACAGACTACTATAACTTTGAACGTATGAATTTGCC**A**AA**A**GATCA**T**CC**T**GCACGTGACATGCAAGATACTTTCTACATTACCGAAGAAATCTTGCTTCGCACTCATACA**TCA**CC**A**GT**T**CAAGC**A**CGTACACTTGATAAACATGATTTTTCTAAAGGTCCTCTTAAGATGATCTCACCAGGACGTGTTTTCCGTCGTGATAC**T**GA**C**GATGC**T**ACTCACAGCCACCAGTTTCACCAAATCGAAGGTTTGGTCGTTGGTAAAAACATCTCAATGGGTGAT**T**TGAA**A**GG**T**AC**A**CTTGAGATGATTATTCAAAAAATGTTTGGTGCAGAACGTCGAATCCGTTTGCGTCCTTCTTACTTCCCATT**TTCA**GAACC**A**TCCGTTGAGGTTGACGTGTCATGCTTCAAGTGTGGTGGTAAAGGATGTAACGTATGCAAGAATACAGGTTGGAT**C**GA**A**AT**T**CTTGGTGCTGGTATGGTTCACCCACAAGTGCTTGAGATGTCAGGTGTTGATTCTGAAGAATATTCA**GGT**TTCG**GT**TT**C**GGT**TTG**GGTCA**G**GAACG**C**AT**C**GC**T**ATGCT**CAGA**TA**C**GG**A**AT**T**AA**T**GA**T**AT**TAGA**GG**A**TT**T**TA**C**CA**G**GG**T**GA**T**GT**A**CG**T**TT**T**TC**T**GA**G**CA**A**TT**C**AA**G**TAA

**Supplementary Figure S4.** **Sequence of *epheS* gene from *S. thermophilus* ST6.** With blue colour are highlighted all altered bases which generate silent mutations to minimize homologous recombination with the genomic copy of *pheS*. With red colour are depicted the bases altered to introduce the T260S and A314G amino acid mutations to the encoded protein, which improve enzyme activity and specificity towards PCPA [2]. Mutations were introduced based on the codon usage of *S. thermophilus* CNRZ1066 (Codon Usage Database at kazusa.or.jp/codon/), with the most frequent codon variant selected for each triplet.


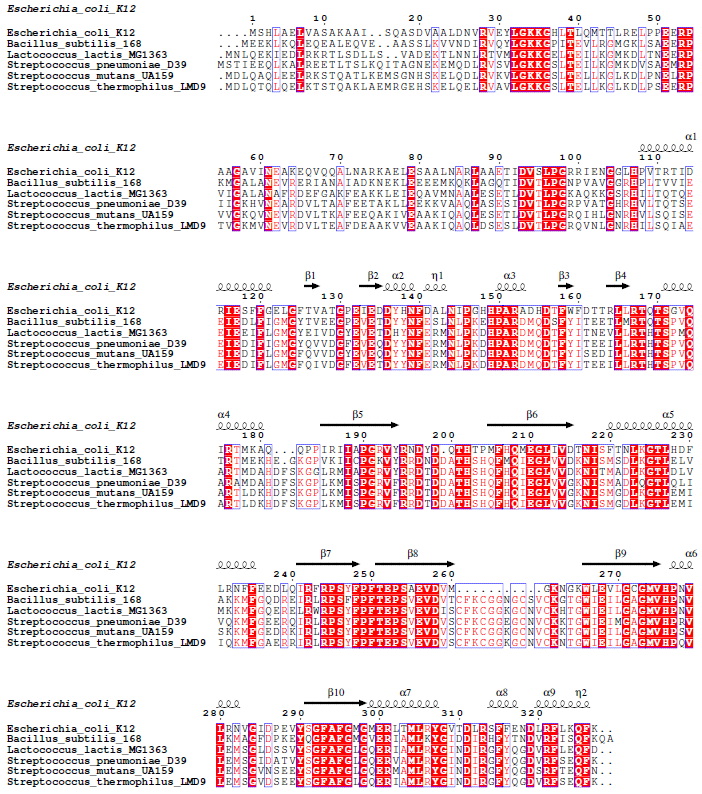


**Supplementary Figure S5. Alignment of the alpha subunit of PheS from different microorganisms.** Alignment was performed using Clustal Omega [3] and visualized using ESPript 3.0 [4]. The depicted secondary structure is derived from the Protein Data Bank entry 6OZ5 [5].

**References**

1. Rau MH, Gaspar P, Jensen ML, Geppel A, Neves AR, Zeidan AA: **Genome-scale metabolic modeling combined with transcriptome profiling provides mechanistic understanding of *Streptococcus thermophilus* CH8 metabolism**. *Appl Environ Microbiol* 2022, **88**.

2. Miyazaki K: **Molecular engineering of a PheS counterselection marker for improved operating efficiency in *Escherichia coli***. *Biotechniques* 2015, **58**:86–88.

3. Sievers F, Wilm A, Dineen D, Gibson TJ, Karplus K, Li W, Lopez R, McWilliam H, Remmert M, Söding J, et al.: **Fast, scalable generation of high-quality protein multiple sequence alignments using Clustal Omega**. *Mol Syst Biol* 2011, **7**:539.

4. Robert X, Gouet P: **Deciphering key features in protein structures with the new ENDscript server**. *Nucleic Acids Res* 2014, **42**:W320–W324.

5. Baidin V, Owens TW, Lazarus MB, Kahne D: **Simple secondary amines inhibit growth of gram-negative bacteria through highly selective binding to phenylalanyl-tRNA synthetase.** *J Am Chem Soc* 2021, **143**:623–627.
